# Incremental Peritoneal Dialysis Favourably Compares with Hemodialysis as a Bridge to Renal Transplantation

**DOI:** 10.4061/2011/204216

**Published:** 2011-09-15

**Authors:** Alessandro Domenici, Maria Cristina Comunian, Loredana Fazzari, Francesca Sivo, Angela Dinnella, Barbara Della Grotta, Giorgio Punzo, Paolo Menè

**Affiliations:** ^1^Nephrology and Dialysis Unit, Department of Cardiovascular, Renal and Pulmonary Diseases, Sant'Andrea Hospital, Sapienza University of Rome, 00189 Rome, Italy; ^2^Peritoneal Dialysis Regional Referral Centre, Nephrology and Dialysis Unit, Civic Hospital, 00042 Anzio, Italy

## Abstract

*Background*. The value of incremental peritoneal dialysis (PD) as a bridge to renal transplantation (Tx) has not been specifically addressed. *Methods*. All consecutive Stage 5 CKD patients with at least 1 year predialysis followup, starting incremental PD or HD under our care and subsequently receiving their first renal Tx were included in this observational cohort study. Age, gender, BMI, underlying nephropathy, residual renal function (RRF) loss rate before dialysis and RRF at RRT start, comorbidity, RRT schedules and adequacy measures, dialysis-related morbidity, Tx waiting time, RRF at Tx, incidence of delayed graft function (DGF), in-hospital stay for Tx, serum creatinine at discharge and one year later were collected and compared between patients on incremental PD or HD before Tx. *Results*. Seventeen patients on incremental PD and 24 on HD received their first renal Tx during the study period. Age, underlying nephropathy, RRF loss rate in predialysis, RRF at the start of RRT and comorbidity did not differ significantly. While on dialysis, patients on PD had significantly lower epoetin requirements, serum phosphate, calciumxphosphate product and better RRF preservation. Delayed graft function (DGF) occurred in 12 patients (29%), 1 on incremental PD and 11 on HD. Serum creatinine at discharge and 1 year later was significantly higher in patients who had been on HD. *Conclusions*. In patients receiving their first renal Tx, previous incremental PD was associated with low morbidity, excellent preservation of RRF, easier attainment of adequacy targets and significantly better immediate and 1-year graft function than those observed in otherwise well-matched patients previously treated with HD.

## 1. Introduction

The feasibility and safety of incremental peritoneal dialysis (PD) as a first-choice renal replacement therapy (RRT) and the good clinical outcome it offers to well-motivated stage 5 CKD patients have been extensively reported [1–4]. This strategy involves elective timely start of PD with a low dose, gradually increased afterwards to compensate ongoing individual residual renal function (RRF) loss to meet total (peritoneal plus residual renal) recommended small solutes clearances adequacy targets [5]. It appears to be a suitable home-based RRT modality, less intrusive on patient's social and active work schedules, which promotes RRF preservation, vascular access sparing, and cost saving. After the very encouraging extension of our preliminary experience [3], this approach has become the standard of practice at our institutions, enabling us to assess the value of incremental PD as a bridge to renal transplantation, an issue that has not been specifically focused upon to date.

## 2. Subjects and Methods

All consecutive incident stage 5 CKD patients attending our advanced uremia clinic for at least one year, who then started RRT with incremental PD or hemodialysis (HD) under our care and subsequently received their first renal transplant (Tx) from January 2000 to December 2008, were included in this observational cohort study. Age, gender, underlying nephropathy, residual renal function (RRF, half-sum of urea and creatinine urinary clearances) and RRF loss rate before dialysis, comorbidities, RRT schedules, adequacy targets (average of quarterly determinations of renal and dialytic urea KT/V, Hb, epoetin weekly dose, serum calcium and phosphate), dialysis-related morbidity, Tx waiting time, RRF at Tx, incidence of delayed graft function (DGF, defined as need of dialysis in the first week after Tx), in-hospital stay for Tx, and serum creatinine at discharge and one year later were recorded. Data were collected and summarized as means ± SD, median ± interquartile range and proportions; continuous variables were compared with the Student's *t-*test for independent samples when normally distributed, or otherwise by the Mann-Whitney test; dichotomous variables were compared with the two-tailed Fisher's exact test; relative risk (RR) with 95% confidence interval (CI) for anuria (urine volume <100 mL/24 hours) at Tx and DGF was calculated and compared between patients on incremental PD or HD before Tx.

## 3. Results

Three hundred and twenty-seven patients with stage 5 CKD who attended the advanced uremia clinic at our institutions electively started RRT under our care in the study period. Modality choice in the whole cohort was determined by patient's preference in 78% of the cases; in 16% of the cases, HD was preferred due to contraindications for PD; in 6% of the cases, PD was chosen because of exhausted vascular access option. The percentage of patient on PD was 21.1% overall. In all 106 subsequently wait-listed patients, RRT modality selection was a patient's choice; 30% of them choose incremental PD. All the patients were followed up by the same nephrology team while on RRT. Those without obvious contraindications were then evaluated for renal transplant suitability by one experienced nephrologist (M. C.Comunian), who was responsible for listing and pre- and post-Tx followup. During the study period, 59 patients received a renal Tx; of these, 18 were not included in this study because of unavailability of predialysis followup (*n* = 5), Tx other than first (*n* = 6), combined kidney-pancreas Tx (*n* = 5), or early Tx failure due to recurrent or *de novo* glomerular disease (*n* = 2). Among included patients, 17 on incremental PD and 24 on HD received a renal Tx after 28 ± 13 and 32 ± 13 months of RRT, respectively (*P* = 0.2); all grafts were from deceased donors, except two from living related donors in 2 patients on HD. Twenty-six patients (63%) were transplanted in one of our regional centers, the remainder in 7 other national referral centers, with a similar distribution of PD- and HD-treated patients. The demographic and clinical characteristics of the study population at the start of RRT are summarized in Table 1. 

Apart from a slightly higher female prevalence among patients on incremental PD, no other differences were found in age, body-mass index, comorbidities, underlying nephropathy, CKD progression rate, and RRF at the start of RRT between patients on incremental PD or HD. There was no RRT modality switch until Tx. Main adequacy parameters during RRT are shown in Table 2. 

Adequate small solute clearances and haemoglobin (Hb) levels were maintained in both groups of patients, although at the expense of a higher, and rapidly increasing, dose of dialysis (see Supplementary Material available online at doi: 10.4061/2011/204216) and epoetin in patients on HD. Serum phosphate and calcium x phosphate product became significantly higher early in the course of RRT in patients on HD and tended to worsen with time. Serum phosphate was <5.9 mg/dL in 76% of 155 quarterly determinations in patients on incremental PD and in only 5% of 252 determinations in patients on HD; serum calcium x phosphate product was <55 mg^2^/dL^2^ in 82% of 155 quarterly determinations in patients on incremental PD and in 25% of 252 determinations in patients on HD. A highly significant reduction of RRF loss rate from −0.97 ± 0.3 to 0.27 ± 0.4 mL/min/month was observed in patients on incremental PD (*P* < 0.001), which was only marginally the case in patients on HD (from −0.99 ± 0.48 to −0.77 ± 0.5 mL/min/month, *P* = 0.06). At the time of Tx, 6 out of 17 patients on incremental PD against 19 out of 24 patients on HD were anuric (*P* = 0.0086, RR 0.44, 95% CI 0.22 to 0.87, *P* = 0.019) (see Supplementary Material). Dialysis-related morbidity was infrequent in this positively selected cohort of CKD patients, being limited to ten peritonitis episodes in 8 patients on PD—corresponding to a cumulative incidence of 0.25 episode per year at risk—and 4 vascular access revascularization procedures in 3 patients on HD. The immediate and medium-term outcome of renal Tx in this cohort is depicted in Table 3.

Time on RRT before Tx was slightly, but not significantly, shorter for patients on PD; 12 patients, one on incremental PD and 11 on HD, suffered DGF, needing three to eleven dialysis sessions (median 6) after TX surgery; overall in-hospital stay for Tx tended to be longer for patients on HD. At discharge, serum creatinine was significantly higher in previously HD-treated patients, as it was one year later. While all PD catheters had been removed within 16 weeks after Tx (3 at the time of Tx surgery), 5 HD patients experienced mildly symptomatic spontaneous thrombosis of the native arteriovenous fistula (AVF), the remainder still harbouring a functional one after a mean followup of 46 ± 28 months. At last followup (48 ± 32 months for patients previously on PD, 41 ± 36 months for those previously on HD), 3 previously HD-treated patients had returned to dialysis because of graft failure after 15, 28, and 43 months, respectively. No patients previously on PD has to date returned to RRT because of graft failure.

## 4. Discussion

Even though when and how to start RRT is still a matter of ongoing investigation and debate, in nonseverely uremic CKD patients electively starting dialysis, the incremental approach seems rationale and would be the preferred choice [5, 6]. In this respect, incremental PD has some inherent and logistical advantages over in-centre HD, being a usually self-performed, home-based therapy, easier to accommodate patient's previous social and active work schedules, which promotes vascular access sparing, longer retention of any clinically relevant RRF, and health care costs saving [3, 5]. Most recent large observational studies comparing outcome of patients starting PD or HD as their first RRT reported a time-dependent advantage in favour of PD during the first 1 to 3 years [7], which overlaps with the waiting time for a deceased-donor Tx in this study. It may be thus reasonably expected that patients scheduled to be timely transplanted could benefit the most from a “PD-first” policy. Furthermore, a low incidence of DGF in patients on PD at the time of Tx has been consistently reported in most large, registry-based studies [8–10] and found to portend a favourable impact on long-term graft and patients outcomes [11, 12]. Our study demonstrated that such results, and perhaps even better ones, can be achieved with the incremental PD-dose strategy. Throughout their time on RRT, our patients on incremental PD showed a more desirable biochemical profile and cost/effective attainment of adequacy targets than their HD counterpart, and we strongly believe that better preservation of RRF is a key factor here. In carefully managed patients on HD, a RRF loss rate similar to that commonly seen with PD has been reported using biocompatible membranes [13] and an incremental dialysis approach [14]. Unfortunately, this was not the case in our patients, despite careful avoidance of overzealous ultrafiltration and nephrotoxic injuries, and the universal use of biocompatible (mainly polysulfone) membranes. Early Tx outcome was dramatically better in PD patients in this study, even more than previously reported [8–10, 15], but we acknowledge that the quite disappointing outcome of some of our HD patients magnified comparison in favour of PD. DGF incidence and 1-year graft function, both regarded as predictive of long-term graft and patient's outcomes [10–12], were substantially worse in our HD patients, despite adopting the suggested policy of avoiding HD, and especially “aggressive” ultrafiltration, in the 24 hours preceding Tx [16]. We present data with the most commonly used dialysis-based definition of DGF, which has been criticized [17], but even using “functional” rival definitions with possibly better predictive power, the results in favour of patients on PD remain significant in ours as well as other studies [8, 18]. 

A further condition recently recognized to negatively affect outcome of patients on RRT and consistently reported to be more frequent in HD patients is pulmonary hypertension [19–21], which appears to be detrimental even with regard to renal Tx [22, 23]. Interestingly enough, it was found in 23% and 54% of our Tx-listed patients on PD and HD, respectively, (*P* = 0.016), reversed after Tx in all but three patients previously on HD and developed *de novo* in a fourth. All these patients have had DGF, and their 1-year graft function was significantly worse than the rest of the HD cohort. Pulmonary artery pressure, either before or after Tx, inversely correlated with graft function in the whole RRT cohort (data not showed). While no high level of evidence-based guidelines on what to do with the AVF in successfully transplanted patients exists [24], some attendant morbidity cannot be excluded [25] and this issue may represent a further overlooked argument in favour of PD prior to renal Tx [26]. 

The present study has of course some obvious limitations; first of all the small number of patients included, which induces caution in extrapolating results to larger populations. The second is our inability to collect sufficient data for a meaningful control of some donor and graft-related factors presently regarded as having an impact on early graft function, such as donor age, gender and cause of death, cold and warm ischemia time, HLA mismatches, and peak of PRA. Even if we cannot formally exclude a bias due to the above-mentioned factors, systematic by chance clustering of unfavourable ones in patients on HD (and/or of favourable ones in patients on PD), large enough to substantially affect results, is quite unlikely. We acknowledge that our results do not apply to pediatric patients and might have been different with a shorter or longer Tx waiting time [27, 28]. Restricting the analysis to patients who wait less than the median actual time in this cohort of patients, however, did not abolish significance in favour of incremental PD, while, on the other hand, our Tx-listed patients on incremental PD experienced an excellent technique survival, with only 2 out of 32 (6.25%) switched to HD because of PD failure after a median followup of 52 ± 23 months. Recently published, large, registries-based studies did not show PD to jeopardise the outcome of dialysis patients remaining on the Tx waiting list, with the only possible exception of those with the higher BMI [29, 30].

We conclude that, in this cohort of patients on RRT receiving their first renal Tx, previous incremental PD was associated with low morbidity, excellent preservation of RRF, easier and more cost/effective management of uremia, and significantly better immediate and 1-year graft function than those observed in otherwise well-matched patients previously treated with HD. According to these results, we believe that the incremental PD option should be offered to every suitable stage 5 CKD patients who appears to be a good candidate for renal Tx. We are committed to improve the early outcome of Tx in our patients on HD.

## Supplementary Material

Supplementary Material: This is to show the observed different course of residual renal function (RRF) that motivated a steeper increase of dialysis dose in patients on hemodialysis until transplant (Tx)

## Figures and Tables

**Table 1:** Demographic and clinical characteristics of the patients.

	Gender	Age, years	BMI, kg/m^2^	Underlying renal disease	RRF loss rate in predialysis, mL/min/month	RRF at dialysis initiation, mL/min
Incremental PD (*n* = 17)	9 F/8 M (53%)	37 ± 13	23 ± 2	3 Alport	−0.97 ± 0.34	6.9 ± 1.1
				2 FSGS		
				2 VUR		
				2 Ig AGNF		
				1 SLE		
				1 MC GNF		
				1 undefined GNF		
				1 DN		
				1 vascular disease		
				3 unknown		

HD (*n* = 24)	10 F/14 M (42%)	43 ± 14	23 ± 2	3 Ig A GNF	−0.99 ± 0.48	6.8 ± 1.5
				3 undefined GNF		
				2 MN		
				2 VUR		
				2 HUS		
				2 Alport		
				1 ADPKD		
				1 DN		
				1 FSGS		
				1 vasculitis		
				6 unknown		

*P* value	0.7	0.1	0.8		0.48	0.4

FSGS: focal segmental glomerulosclerosis; VUR: vescicoureteral reflux; SLE: systemic lupus erythematosus; MC GNF: mesangiocapillary glomerulonephritis; DN: diabetic nephropathy; MN: membranous nephropathy; HUS: haemolytic uremic syndrome; ADPKD: autosomal dominant polycystic kidney disease; GNF: glomerulonephritis; Ig A: immunoglobulin A.

**Table 2:** Main adequacy parameters during RRT.

	Weekly dialytic urea KT/V at start	Weekly dialytic urea KT/V at Tx	Time-averaged Hb, g/dL	Time-averaged EPO dose, U/Kg/week	Time-averaged Ca x P, mg/dL	Time-averaged serum phosphate, mg/dL	RRF loss rate, mL/min/month
Incremental PD (*n* = 17)	0.69 ± 0.2	1.33 ± 0.3	11.9 ± 0.9	112 ± 33	50 ± 4	5.7 ± 0.4	−0.27 ± 0.4
HD (*n* = 24)	2.5 ± 0.5*	3.3 ± 0.3*	12.3 ± 0.9	199 ± 58	58 ± 4	6.8 ± 0.4	−0.77 ± 0.5
*P* value	<0.0001	<0.0001	0.2	<0.0001	<0.0001	<0.0001	<0.0001

*Daugirdas single-pool.

**Table 3:** Main TX outcome data.

	Tx waiting time, months	In-hospital stay for Tx, days	DGF *n *	Serum creatinine at discharge, mg/dL	Serum creatinine at 3 months, mg/dL	Serum creatinine at 1 year, mg/dL
Incremental PD (*n* = 17)	26 ± 15	19 ± 4	1	1.3 ± 0.3	1.2 ± 0.3	1.14 ± 0.3
HD (*n* = 24)	28 ± 20	22 ± 6	11	2.1 ± 0.9	1.6 ± 0.5	1.96 ± 0.9
*P* value	0.2	0.08	0.006	0.0013	0.0031	0.0016

DGF: delayed graft function.
