# Supplementary material for: Incremental Peritoneal Dialysis Favourably Compares with Hemodialysis as a Bridge to Renal Transplantation
Source: Int J Nephrol. 2011 Sep 15;2011:204216. doi: 10.4061/2011/204216 (PMC3173956; doi:10.4061/2011/204216)
Supplement: Supplementary file 1 — Supplementary Material: This is to show the observed different course of residual renal function (RRF) that motivated a steeper increase of dialysis dose in patients on hemodialysis until transplant (Tx) [file 204216.f1.pdf]

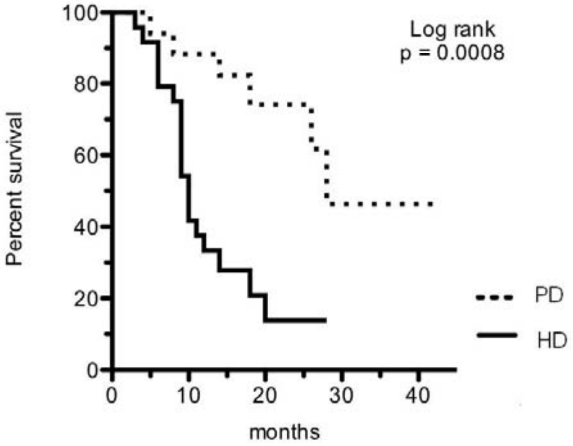

Fig 1 Tx-censored Kaplan-Meyer survival of significant RRF (>100 ml/day) during incremental PD or HD

	at RRT initiation (number of patients)		at Tx	
PD				
	2 liters once daily	(5)	2 liters twice daily	(3)
	2 liters twice daily	(5)	2 liters thrice daily	(2)
	10 liters nightly APD	(5)	2 liters 4 times daily	(1)
	15 liters nightly APD	(2)	10 liters nightly APD	(1)
			15 liters nightly APD	(4)
			20 liters nightly APD	(4)
			25 liters nightly APD	(1)
			22 liters CCPD	(1)
HD				
	3 hours twice weekly	(5)	3 hours thrice weekly	(1)
	3,5 hours twice weekly	(3)	3,5 hours thrice weekly	(4)
	3 hours thrice weekly	(6)	4 hours thrice weekly	(17)
	3,5 hours thrice weekly	(8)	4 hours 4 times a week	(2)
	4 hours thrice weekly	(2)		

Table 4 Dialysis dose at the beginning of RRT and at transplant time
